# An overview of mast cell pattern recognition receptors

**DOI:** 10.1007/s00011-018-1164-5

**Published:** 2018-06-16

**Authors:** Justyna Agier, Joanna Pastwińska, Ewa Brzezińska-Błaszczyk

**Affiliations:** 1 Department of Experimental Immunology, Medical University of Lodz, Pomorska 251, 92-213 Lodz, Poland; 2 Laboratory of Cellular Immunology, Institute of Medical Biology, Polish Academy of Sciences, Lodz, Poland

**Keywords:** Mast cells, PRRs, TLRs, CLRs, NLRs, RLRs.

## Abstract

**Background:**

Mast cells (MCs) are long-lived immune cells of the connective tissue which play a key role in development and amplification of inflammatory process initiated inter alia by allergic reactions or microbial infections. They reside in strategic locations in the body that are notably exposed to deleterious factors disturbing homeostasis, which enables them to become one of the first-line defense strategy. MCs have developed a wide range of various mechanisms to deal with invading intruders and harmful endogenic factors. Those include storage and synthesis with a subsequent release of inflammatory mediators, forming of MC-extracellular traps, and phagocytosis.

**Findings:**

Particularly, important role in microbial sensing is achieved due to the presence of different pattern recognition receptors (PRRs). The best-described receptors are Toll-like receptors activated by different pathogen- and damage-associated molecular patterns. However, MCs express also C-type lectin receptors specialized in antifungal defense, NOD-like receptors detecting bacterial peptidoglycans, and RIG-like receptors relevant in viral sensing.

**Conclusion:**

This review will focus on the current knowledge of PRRs expressed within different types of MCs.

## Mast cells

For a long time, mast cells (MCs) have been recognized as the critical immune system weapon in the allergic reactions [1, 2], as well as during parasitic infections [3]. Indeed, these immune system’s chief sentinels hold a multitude of functions in the host defense against a variety of microorganisms, mainly bacteria and viruses [4–7]. So far, the contribution of MCs to the protection against fungal infections is an almost unexplored area [8]. MCs are long-lived resident cells located preferentially throughout vascularized connective tissues. They are numerous at the junction point of the host and external environment, including the skin, the respiratory system, the gastrointestinal and genitourinary tracts; therefore, they belong to the first immune cells, which encounter intruders [9]. Since MCs are rich source of numerous biologically active mediators, such as granule-associated preformed mediators (e.g., neutral proteases, histamine, proteoglycans, metalloproteinases), de novo generated arachidonic acid metabolites (e.g., leukotrienes, prostaglandins, thromboxanes), as well as many newly synthesized cytokines and chemokines, they can alter the functions of the surrounding cells and tissues. MC-derived proinflammatory mediators, cytokines and chemokines induce the development of inflammation at the site of pathogen entry [10–14]. MCs are highly efficient effector cells that release not only active mediators but also antimicrobial peptides, such as cathelicidins [15]. Moreover, these cells can phagocytose and subsequently kill bacteria, via oxidative and non-oxidative systems [16, 17]. MCs also contribute to host defense by forming extracellular traps (MCETs) composed of DNA, histones, and granule proteins, which can entrap and kill various microbes [18]. Furthermore, following phagocytosis these cells have the capability of processing bacterial antigens for presentation through class I and II MHC molecules, which leads to the development of adaptive antimicrobial immunity [17, 19, 20].

To respond rapidly to infection or cellular damage, MCs express germline-encoded pattern recognition receptors (PRRs) that recognize unique bacterial, viral, fungal or parasitic components known as pathogen-associated molecular patterns (PAMPs) and host-derived molecules, called damage-associated molecular patterns (DAMPs). Hitherto, PRRs have comprised five families: Toll-like receptors (TLRs), C-type lectin-like receptors (CLRs), retinoic acid-inducible gene I (RIG-I)-like receptors (RLRs), nucleotide-binding oligomerization domain (NOD)-like receptors (NLRs) (Table 1), and recently designated absent-in-melanoma (AIM)-like receptors (ALRs). While TLRs constitute the best characterized PRRs in MCs, recent findings suggest that other types of recognition receptors act as surveillance molecules in these cells. In this review, we will provide a concise overview of the TLR, CLR, RLR, and NLR family members in MCs.

**Table 1:** Members of the TLR, CLR, NLR and RLR families with their alternative names (separated by forward slashes), divided into relevant subgroups or subfamilies

Type	Family member		
TLRs	TLR1-10 TLR11-13		
CLRs	Group I	MR/CD206 DEC205/LY75/CD205	
	Group II	DC-SIGN/CD209 langerin/CLEC4K/CD207 MGL/CLEC10A/CD301 CLEC5A/MDL1	
		Dectin 1 subfamily	Dectin 1/CLEC7A MICL/CLEC12A/DCAL2 CLEC2/CLEC1B DNGR1/CLEC9A CLEC12B
		DCIR subfamily	DCIR/CLEC4A Dectin 2/CLEC6A BDCA2/CLEC4C/CD303 Mincle/CLEC4E
NLRs	NLRA	CIITA	
	NLRB	NAIP	
	NLRC	NLRC1/NOD1 NLRC2/NOD2 NLRC3-5 NLRX1	
	NLRP	NLRP1 NLRP2 NLRP3 NLRP4-14	
RLRs	RIG-I MDA5 LGP2		

The underlined receptors are those that have been documented in MCs

## Toll-like receptors (TLRs)

### TLR family, their ligands and molecular structure

TLRs are the best studied and described molecules among other PRRs. They comprise a large group of transmembrane receptors expressed in various types of cells including hepatocytes [21], monocytes/macrophages [22], dendritic cells (DCs) [22, 23], B lymphocytes [24], T lymphocytes [25], epithelial cells [26], endothelial cells [27], neutrophils [28], and NK cells [29]. Most importantly, TLRs are expressed in MCs [30, 31]. The TLR family include TLR1, TLR2, TLR3, TLR4, TLR5, TLR6, TLR7, TLR8, TLR9, TLR10, TLR11 [32], TLR12 [33] and TLR13 [34]. Hitherto sources strengthen the conviction that certain TLRs are present merely in a particular membrane structure, e.g., cellular membrane and endosome membrane [32]. TLRs recognize a wide range of molecular patterns associated with bacteria, viruses, protozoa or parasites, as well as different endogenous factors arising as a result of any tissue damage [32, 35]. Various members of TLR family may sense the same molecular patterns or entirely different, depending on the type of TLR. Mentioned ligands include lipoproteins, diacyl and triacyl lipoproteins, lipoteichoic acid (LTA), lipopolysaccharide (LPS), single- and double-stranded RNA, unmethylated CpG oligodeoxynucleotide DNA, flagellin, heat shock protein (HSP)70, profilin-like molecule, myeloid differentiation factor 2 (MD2), oxidized phospholipids, fibrinogen, and many others [30–32]. TLRs are composed of the N-terminal ligand recognition domain, transmembrane region and a C-terminal cytoplasmic signaling domain exhibiting homology of a domain present in interleukin (IL) type 1 receptor (IL-1R). Thus, it is called Toll IL-1R (TIR) domain. TIR domains are a part of adaptor proteins and play a crucial role in the early phase of signaling pathways. Transmembrane domains are responsible for the interaction with multispan transmembrane protein Unc-93 homolog B1 (UNC93B1), resulting in trafficking of TLRs within endocytic compartments of the cell. TLRs which transmembrane domains do not bind to UNC93B1 are directed to the surface of the cell. N-terminal ectodomains (ECDs), built of leucine-reach repeat (LRR) motif, constitute glycoproteins that recognize molecular patterns associated with pathogens [36].

### TLR signaling pathways

Recognition of any inducing agents by TLRs leads to expression of different genes accordingly to the type of cell and TLR engaged in this process. TLR signaling pathway is usually based on two different mechanisms, which depend on TIR domain-containing adaptors that are recruited to TLRs, namely myeloid differentiation primary response gene 88 (MyD88) and TIR domain-containing adaptor-inducing interferon (IFN)-β [TRIF or Toll-like receptor adaptor molecule 1 (TICAM-1)]. However, there are three other adaptor molecules, that may be required for bridging and activating MyD88 or TRIF pathways: TIR domain containing adaptor protein/MyD88-adapter-like (TIRAP/Mal), TRIF-related adaptor molecule (TRAM) and Sterile-alpha and Armadillo motif-containing protein (SARM). Various TLRs, but not TLR3, may initiate the MyD88 signaling pathway. Moreover, in the case of particular TLRs, e.g., TLR1, TLR2, TLR4 and TLR6, MyD88 signaling is preceded by TIRAP/Mal pathway. TLR3 and TLR4 may initiate the TRIF signaling pathway, however, in case of TLR4, this process is preceded by TRAM signaling. The signaling cascade leading to the activation of nuclear factor kappa B (NF-κB) results in the expression of various cytokines in a cell engaged in the entire process, while activation of IFN regulatory factor (IRF) 3 or IRF7 leads to the expression of type I IFNs [32]. The signal cascade initiated by the activated TLR is complex, which makes it possible to regulate this process in many stages. A recent report has shown that leukotrienes C4 and D4 have a considerable influence on the down-regulation of TLR1–7 and this effect is mediated by activation of the CysLT1 receptor [37]. Karpov et al. have hypothesized that inflammatory GPCR signaling (via CysLTR) interacts with and modifies mast cell TLR expression and activation pathways, ultimately blocking activation of NF-κB.

### TLRs in MCs

Indisputably, all the studies concerning the presence of TLRs in MCs carried out to date confirm the expression of various members of the TLR family within different types of MCs to a greater or lesser degree. The expression of TLRs in MCs has been confirmed both at mRNA and protein level in a wide range of MCs including murine MCs, e.g., murine bone marrow-derived MCs (BMMCs), murine fetal skin-derived MCs (FSDMCs), murine peritoneal cell-derived MCs (PCMCs); rat peritoneal MCs (RPMCs); human MCs comprising immature human MCs, e.g., human BMMCs, cord blood-derived MCs (CBMCs), and mature human MCs, e.g., MCs isolated from human lungs, MCs isolated from human skin; human MC lines, e.g., human MC line-1 (HMC-1), laboratory of allergic diseases 2 MCs (LAD2); as well as mouse MC lines, e.g., MC/9, P815. Data indicate that MCs express TLR1–10 and there is no information confirming the presence of TLR11–13. Additionally, our recent data revealed that nearly every TLR might be expressed either in MC plasma membrane as well as inside the cell [38, 39]. More detailed data concerning this issue are already described in other reviews [30, 31].

## C-type lectin receptors

### CLR family, their ligands and molecular structure

CLRs constitute a group of PRRs that play a pivotal role in antifungal defense strategies and may cooperate with other PRRs, such as TLRs, in various immunological mechanisms [40]. CLRs are expressed in different cell types including endothelial cells [41] and DCs [42, 43]. Additionally, their presence is noticed in myeloid cells, e.g., neutrophils [44], monocytes/macrophages [45], and Langerhans cells [42]. Current knowledge assumes that CLR family is slightly greater and more complex in comparison to TLR members, and consists of two major groups with subgroups of receptors. Group I CLRs includes the Mannose Receptor family with Mannose Receptor (MR or CD206) and DEC205 (LY75 or CD205) as representatives. Group II CLRs comprise asialoglycoprotein receptor family that consists of DC-specific intercellular adhesion molecule-3-grabbing non-integrin (DC-SIGN or CD209), langerin (C-type lectin domain family (CLEC) 4 member K or CD207), macrophage galactose-type lectin (MGL, CLEC10A or CD301) and CLEC5A [myeloid DAP12-associating lectin 1 (MDL1)]. Moreover, group II CLRs includes two subgroups, namely Dectin 1 subfamily and DC immunoreceptor (DCIR) subfamily. The former one consists of Dectin 1 (CLEC7A), myeloid C-type lectin-like receptor [MICL, CLEC12A or DC-associated lectin 2 (DCAL2)], CLEC2 (CLEC1B), DC NK lectin group receptor 1 (DNGR1 or CLEC9A) and CLEC12B, while the latter one comprise DCIR (CLEC4A), Dectin 2 (CLEC6A), blood DC antigen 2 (BDCA2, CLEC4C or CD303) and macrophage inducible Ca^2+^-dependent lectin (Mincle or CLEC4E) [46]. CLRs are associated with membranes, and they constitute a family of transmembrane signaling proteins [32]. Those receptors are capable of sensing various carbohydrate molecules present in cell walls of fungi. The vast majority of them may bind to mannose and fucose sugars, while some of them can also recognize sulphated sugars, β-1,3-glucan (in the case of Dectin receptors) and spliceosome-associated protein 130 (SAP130, for Mincle receptor) [32, 46, 47]. CLRs possess one or more carbohydrate-recognition domains (CRDs), that act in a calcium-dependent manner, sensing and binding sugar molecular patterns by ligation to Ca^2+^. CRDs form compact and spherical structures comprising conserved regions, which is responsible for CLRs affinity to specific carbohydrates. However, some CLRs such as Dectin 1 can bind carbohydrates in a Ca^2+^-independent manner due to the presence of non-canonical sugar binding site [46, 48]. Certain CLRs also possess immunoreceptor tyrosine-based activation motif (ITAM) domains or immunoreceptor tyrosine-based inhibition motif (ITIM) domains or do not clearly express any of this domain within cytoplasmic tail [49].

### CLR signaling pathways

CLRs signaling pathway is directly dependent on the presence or absence of ITAM or ITIM domains. Receptors such as DNGR1, Dectin 1, Dectin 2, Mincle and CLEC5A expressing ITAM domain can initiate a signaling cascade through spleen tyrosine kinase (Syk). Moreover, DNGR1 and Dectin 1 activate Syk *via* direct interaction with ITAM domain, while others recruit Syk by ITAM-associated adaptor molecules, e.g., Fc receptor γ-chain (FcRγ) or DAP12 [32, 49]. Syk activation leads to a formation of caspase recruitment domain-containing protein 9 (CARD9), mucosa-associated lymphoid tissue lymphoma translocation protein 1 (Malt1) and B cell lymphoma 10 (Bcl10) complex, which in turn triggers mitogen-activated protein kinases (MAPK) and NF-κB. In consequence, this signaling pathway results in initiation of pro-inflammatory processes such as synthesis of mediators, chemotaxis, phagocytosis, and activation of inflammasomes. DCIR express ITIM domain is interacting via Src homology region 2 domain-containing phosphatase (SHP)-1 and SHP-2 that is responsible mainly for inhibition of mediator production initiated by different PRR, e.g., TLR8, TLR9. Receptors without ITAM or ITIM domains include MR, DEC205, and DC-SIGN. While in the case of MR and DEC205 there is no clearly defined mode of a signaling pathway, DC-SIGN, as well as Dectin 1, are able to induce pro-inflammatory processes independently from Syk via Raf1 Proto-Oncogene [40, 47, 49].

### CLRs in MCs

Data showing the expression of CLRs within MCs, unlike TLRs presence, are still limited. Furthermore, among investigated receptors from the CLR family, the most commonly presented studies concerned Dectin 1, and only a few data revealed the expression of MR, MGL, and Mincle within MCs. Thus, this issue is yet to be explored. Out of various MCs and their cell lines, murine BMMCs were used predominantly to investigate the presence of CLRs within MCs, however murine PCMCs, RPMCs, peripheral blood-derived MCs (PBMCs), and human CBMCs were studied as well. Moreover, human KU812 and rat RBL-2H3 basophils cell lines were the subject of research for those studies, since they share certain characteristics with MCs, e.g., MC IgE-mediated degranulation. mRNA expression of different CLRs within MCs was studied exclusively by one method, namely RT-PCR, sometimes also carried out with real-time analysis, RT-qPCR, and in one case even confirmed with RNA direct sequencing. Unlike mRNA, various CLR protein expression in MCs was investigated using more techniques and tools including Western blot, immunohistochemistry, immunoblotting, fluorescence-activated cell sorting (FACS) and even ELISA in the sense of checking the secretion of MC cytokines after the use of certain CLR inhibitor [50–56].

Dectin-1 is the best known and described receptor from the whole CLR family. It is responsible for the recognition of β-glucan, comprising the main building block for fungal cell wall, and stimulation of tumor necrosis factor (TNF) and reactive oxygen species (ROS) production. Dectin-1 is expressed within various immune cells including neutrophils, monocytes/macrophages, NK cells, DCs, and fibroblasts, but also within murine BMMCs supplemented with mouse plasma. RT-PCR, with a subsequent product visualization by agarose gel electrophoresis, revealed a low mRNA expression of Dectin-1 isoform A and isoform B in murine BMMCs stimulated with zymosan, a β-glucan from yeast cells. FACS analysis with a Dectin-1 specific antibody showed a weak cell surface expression of Dectin-1 on untreated murine BMMCs and a significantly higher expression in cells stimulated with zymosan. However, this functional protein expression was not dependent on the presence of mouse plasma. Interestingly, cell surface expression of Dectin-1 was significantly lower in cells treated with a Dectin-1 inhibitor, namely laminarin. Furthermore, FACS enabled the measurement of ROS production mediated by Dectin-1, since ROS levels were significantly lower in laminarin-treated cells compared to those untreated [56].

Further studies with another β-glucan, specifically curdlan that constitute an agonist for Dectin-1, indicate the induction of murine BMMC degranulation, including a significant increase of histamine release measured in supernatants by ELISA technique. Moreover, murine BMMCs released from their granules β-hexosaminidase, which levels were estimated by an enzymatic colorimetric assay. Entirely different observations were made in the case of de novo synthesized mediators, such as LTC_4_, IL-6, and CCL2. Analysis of supernatants by ELISA technique revealed that murine BMMCs treated with curdlan did not produce mentioned mediators unless the cells were activated through IgE receptor cross-linking. Interestingly, those mechanisms differ greatly from processes initiated by TLR signaling pathways, where de novo synthesis of lipid mediators and cytokines is triggered independently of degranulation [50].

The presence of Dectin-1 within murine BMMCs and RPMCs was also confirmed through their direct stimulation with *Candida albicans* yeasts or hyphae, which significantly induced murine BMMC and RPMCs degranulation, assessed by β-hexosaminidase release. Analysis of supernatants by ELISA technique also revealed a considerably high level of TNF, which increased even more with time after murine BMMC fungal stimulation compared to untreated cells. Furthermore, *C. albicans* induced production of other cytokines or chemokines including IL-1β in the case of yeasts, IL-6, delayed synthesis of IL-10, CCL3, and CCL4, as well as induced ROS production. To confirm Dectin-1 mediated initiation of cytokine synthesis, Nieto-Patlán et al. [52] treated murine BMMCs with anti-Dectin-1 and measured the release of TNF. They observed a significant decrease in TNF production when compared to murine BMMCs incubated with anti-Dectin-1 isotype control.

Dectin-1 receptor expression was assessed as well within other MCs and MC-like cell types including human CBMCs and human KU812 cell line. mRNA expression of Dectin-1 isoform B obtained through RT-PCR was observed in both untreated MC types, which was also confirmed by direct sequencing that revealed a complete sequence identity of the obtained product when compared to known Dectin-1 isoform B sequence. Likewise, the presence of the functional Dectin-1 isoform B protein was observed by Western blot in human KU812 when treated with IL-4 or sodium butyrate, while not stimulated human CBMCs weakly expressed Dectin-1 isoform B as well as isoform A. Moreover, supernatants from human CBMCs incubated with zymosan and peptidoglycan (PGN) were analyzed for certain cytokine and lipid mediator secretion. Although, the levels of IL-1β, granulocyte–macrophage colony-stimulating factor (GM-CSF), LTB_4_, and LTC_4_ were significantly higher in human CBMCs treated with zymosan and PGN compared with not stimulated cells, only LTC_4_ production was found to be Dectin-1 mediated, since pre-treatment with laminarin and other Dectin-1 inhibitor, glucan phosphate, significantly reduced LTC_4_ release in human CBMCs incubated with zymosan [53].

Ribbing et al. [54] studied the expression of Dectin-1 and, responsible mainly for glucose or mannose sensing, Mincle receptor within human PBMCs isolated from healthy control (HC) and atopic eczema (AE) patients. Low mRNA expression of both receptors obtained through RT-qPCR was observed in untreated PBMCs from HC as well as from AE subjects. A significantly higher expression of those receptors was noticed in PBMCs stimulated *via* IgE receptor cross-linking together with *Malassezia sympodialis* in both groups of patients. MCs activation through IgE receptor cross-linking and incubation with *M. sympodialis* separately, revealed a higher expression of Dectin-1 in HC and a slight increase in mRNA levels in AE patients when compared to not stimulated cells, while increased expression of Mincle was comparable in HC and AE subjects. Western blot analysis confirmed the functional protein expression of both receptors, Mincle and Dectin-1, including Dectin-1 isoform A and isoform B, in PBMCs after IgE receptor cross-linking. Furthermore, biopsy samples taken from lesional HC and AE skin subjects and stained by immunohistochemistry revealed the presence of Dectin-1.

Expression of Mincle mRNA studied by RT-PCR and confirmed by electrophoresis was as well observed in rat MC-like RBL-2H3 cell line. However, due to the lack of commercially available anti-Mincle antibody which would recognize and attach to rat Mincle, any further studies involving assessment of functional Mincle protein presence and levels is not possible, unless the cells are transfected with vectors expressing functional Mincle [51].

Finally, expression of MR, which recognize fucose, mannose or *N*-acetylglucosamine, and MGL, sensing *N*-acetylgalactosamine within particular MCs was studied among various CLRs. The expression of functional proteins of both receptors was confirmed in murine BMMCs and murine PCMCs with the use of FACS. Interestingly, ELISA technique revealed a reduced production of TNF and IL-6 in murine BMMCs and murine PCMCs, both treated with anti-MR or anti-MGL antibodies and *Bordetella pertussis*, and IFN-γ only in case of murine PCMCs, incubated with anti-MR or anti-MGL antibodies and *B. pertussis* when compared to not stimulated cells [55].

## NOD-like receptors (NLRs)

### NLR family, their ligands and molecular structure

The cytosolic nucleotide-binding oligomerization domain receptors (also known as CATERPILLERs) are multi-domain proteins with a tripartite architecture. NOD-like receptors are typically composed of four variable N-terminal effector domains, which are used to subdivide NLRs into four groups: the acidic transactivation domain (subfamily NLRA), the baculoviral inhibitory repeat-like domain (subfamily NLRB), the CARD (subfamily NLRC), and the pyrin domain (subfamily NLRP). The N-terminal domain performs effector functions by interacting with other proteins [57, 58]. The central nucleotide-binding domain NACHT (also referred to as NOD domain) shares homology with the AAA + superfamily of ATPases and is involved in dNTPase activity and oligomerization [58–60]. The C-terminal LRRs are involved in ligand binding or activator sensing [58, 61]. NLRs comprise a large family of 23 members in human and constitute a major group of intracellular PRRs [57]. The MHC-II transactivator (CIITA) is the only member of the NLRA subfamily. Likewise, the NLRB subfamily has only one receptor, NAIP. The NLRC subfamily consists of NLRC1 (NOD1), NLRC2 (NOD2), NLRC3-5, and NLRX1 [57, 58, 61]. The best characterized NLRs, NOD1 and NOD2, were the first receptors from this family identified as sensors of microbial patterns [62, 63]. NOD1 and NOD2 detect distinct motifs of PGN, which is a fundamental component of the bacterial cell wall. NOD1 binds the d-γ-glutamyl-meso-DAP dipeptide (iE-DAP), which is found in all Gram-negative and certain Gram-positive bacteria (*Listeria* sp. and *Bacillus* sp.) [64]. In turn, NOD2 identify the muramyl dipeptide (MDP) structure found widely in nearly all bacteria [65]. Thus, NOD1 is involved in the identification of a specific subset of bacteria and NOD2 acts as a general sensor of PGN. The last subfamily—NLRP consists of 14 members, NLRP1-14 [57, 58, 61]. Interestingly, NLRP1 and NLRP3 induce the formation of the inflammasome complex that stimulates caspase-1 activation to promote the secretion of proinflammatory cytokines [66].

### NLR signaling pathways

The current model assumes that the crucial step in NLR activation is the self-oligomerization of the N-terminal NACHT domain upon recognition of their ligands. This step is followed by the transient recruitment and activation of the CARD-containing kinase RIPK2 (also called RIP2/RICK), a potent activator of NF-κB and MAPKs, through CARD–CARD interaction. Recruitment of RIPK2 leads to NF-κB and the inhibitor of NF-κB-(IκB-) kinase (IKK) complex activation through phosphorylation and ubiquitination of IκBα. It is accomplished via the recruitment of the E3 ubiquitin ligase TRAF6 to RIPK2, followed by TRAF6 autoubiquitination, polyubiquitination of RIPK2, and the ubiquitination-dependent signaling and activation of the TAK1 complex. The activated TAK1 complex promotes the K63-type polyubiquitination of IKK-β, culminating in the degradation of the NF-κB repressor IκB, the translocation of NF-κB to the nucleus, and the transcription of proinflammatory genes. In addition, recruitment of RIPK2 also activates p38, ERK, and JNK, and it seems to be possible that NLRs participate in the activation of the type I IFN pathway. Although, RIPK2 is critical for the induction of NF-κB activation, the molecular basis how the NOD/RIPK2 complex stimulates NF-κB activation is poorly understood [57].

### NLRs in MCs

Few sources indicate that NLRs are expressed in RPMCs, mouse MC line P815, and mouse BMMCs. Zabucchi et al. [67] stated by immunogold analysis that in resting RPMCs, NOD1 and NOD2 receptors are associated with the same subcellular compartments such as surface and matrix of secretory granules or with cytoplasmic vesicles. Additionally, gold particles labeling showed that free cytosolic NLRs in RPMCs are rare. Interestingly, after ingestion of opsonized *Escherichia coli*, the extent of labeling for NOD1 and NOD2 in RPMC granules decreased significantly.

Expression of NOD2 mRNA was observed in P815 cells [68]. Real-time PCR results showed that NOD2 mRNA expression was increased considerably in P815 cells after *Staphylococcus aureus* infection. Furthermore, IL-6, CXCL8 and histamine secretion was significantly up-regulated in the *S. aureus*-infected cells pretreated with NOD2 siRNA before infection. Noteworthy, *S. aureus* infection could trigger NF-κB activation in P815 cells, but NOD2 siRNA treatment significantly reduced the NF-κB activation in response to *S. aureus* and these results may indicate that the NF-κB activation was closely associated with NOD2.

Ueno et al. [69] established transient induction of endogenous NLRP3 protein in BMMCs after stimulation with stem cell factor (SCF) alone or after stimulation via FcɛRI crosslinking by anti-DNP-IgE and DNP-BSA. Moreover, in BMMCs stimulated with the cytokine triad: SCF + IL-1β + IL-10, there was a severe induction of NLRP3 protein expression that continued to increase over 24 h. Persistent induction of NLRP3 protein was preceded by the transient expression of NLRP3 mRNA, which peaked after 2 h and declined after 6 h as assessed by Northern blotting. Authors also examined the expression of other inflammasome-associated genes such as ASC and RIP2, which are known to form a complex with NLRP3. RT-PCR revealed that the expression of RIP2 was increased (as in the case of NLRP3), after stimulation for 2 h with the cytokine triad. Intriguingly, NLRP3 had a negative regulatory role in the cyclooxygenase (COX)-2 dependent production of prostaglandin (PGD)2 by BMMCs.

The expression of NLRs was also confirmed in human CBMCs, MCs from human colon specimens and HMC-1. Enoksson et al. [70] observed by FACS that CBMCs isolated from healthy donors expressed NOD1 and NOD2 protein. All tested individuals expressed NOD1 protein (NOD1^+^ CBMCs: 75.4 ± 8.3%), but only one donor expressed NOD2 receptor at low level (NOD2^+^ CBMCs: 6.6 ± 6.0%). Moreover, NOD1 specific agonist M-TriDAP induced an increase in CXCL8, CCL3, CCL4, and TNF secretion by CBMCs and this effect was mediated through p38 and/or RIP2-dependent pathways. Interestingly, a synergistic effect of the co-stimulation with M-TriDAP in combination with LPS on CXCL8 release by CBMCs was also observed. Analysis of FACS-sorted human CBMCs confirmed the expression of NOD2 and NLRP3, two of the major receptors for MDP [71]. Moreover, the effect of specific NLR agonists on sustained CBMCs activation was also observed. CBMCs were incubated with *S. aureus* PGN, MDP, and murabutide (a synthetic MDP analogue). PGN induced the production of IL-1β, IL-6, and CXCL8 by MCs. Neither NLR activator MDP nor synthetic analogue alone induced detectable MC response elicited by cytokine release. Interestingly, NLR agonists potentiated the Pam_3_CSK_4_-induced MC IL-6 response in a concentration-dependent manner.

Analysis of sequential sections of healthy human colon specimens with anti-tryptase and anti-NOD2 monoclonal antibodies (mAbs) revealed MCs with NOD2 [72]. Moreover, analysis of inflamed colon specimens from Crohn’s disease (CD) and ulcerative colitis (UC) patients revealed that NOD2^+^ MCs were localized in the lamina propria. Interestingly, the analysis of adjacent sections showed that the densities of NOD2^+^ MCs were significantly increased in CD specimens in comparison to UC specimens and controls. The same authors stressed that NOD2 mRNA and protein expression was markedly higher in IFN-γ-treated PBMCs and human lung MCs than in IFN-γ-untreated MCs.

The qPCR results showed that NOD2 mRNA was also detected in HMC-1 [73]. To establish the role of NOD2 in PGN-induced MC activation, HMC-1 were preincubated with either anti-NOD2 antibody or transfected with siRNA-NOD2. As a result, histamine release from pretreated or transfected HMC-1 was significantly inhibited as compared with controls. These results strongly suggest that NOD2 plays a critical role in PGN-induced HMC-1 activation.

## RIG-like receptors (RLRs)

### RLR family, their ligands and molecular structure

RLRs are a family of DExD/H box RNA helicases that function as cytoplasmic sensors of viral RNA. So far, three RLR members have been identified: RIG-I, MDA5 (melanoma differentiation associated factor 5), and LGP2 (laboratory of genetics and physiology 2) [74]. All RLR family members have a central DExD/H box RNA helicase domain with ATPase activity. The C-terminal region consists of a domain termed the RNA recognition domain which is responsible for binding RNA. In addition, the C-terminal domains of RIG-I and LGP2 have been shown to act as repressor domains (RD), ensuring that the receptors remain in an inactive form. Both RIG-I and MDA5 have two N-terminal CARDs, while LGP2 lacks this domain. For this reason, LGP2 is rather considered to be RIG-I and MDA5 signaling regulator than active receptor [74, 75]. Because RLRs detect various types of viral RNA, these molecules are one of the first line of defense against virus infection [76]. RIG-I senses short (< 300 bp) 5′ triphosphorylated dsRNA or ssRNA with double-stranded regions. Additionally, it binds specific dsRNAs lacking a 5′ phosphate or containing a 5′ monophosphate. In turn, MDA5 binds to longer (> 1 kb) dsRNA [77–79].

### RLR signaling pathways

In the RIG-I pathway, RNA binding is modulated by polyubiquitination with TRIM25 and Riplet (RNF135) which act as E3 ubiquitin ligases. They mediate the covalent attachment of the K63-linked polyubiquitin chains. In turn, little is known about the molecules required for the MDA5 polyubiquitination. Following this, the CARDs of RIG-I and MDA5 trigger signaling cascades by interacting with the N-terminal CARD-containing adaptor IFN-β-promoter stimulator 1 (IPS-1/MAVS/CARDIF/VIsA) localized on the mitochondrial membrane. Dimerization of IPS-1 activates TRAF-2, TRAF-6, and TRADD, which recruits TRAF-3 and TRAF family member-associated NF-κB activator (TANK) to trigger the activation of tank-binding kinase-1 (TBK1) and I κB kinase epsilon (IKK-ε). Interestingly, NLRX1 may act as an inhibitor of IPS-1 signaling. In addition to the TRAFs and TRADD, translocase of the outer membrane 70 (TOM70) in the mitochondria and stimulator of interferon genes (STING) localizes to the outer membrane of the endoplasmic reticulum may also be involved in facilitating RIG-I signaling [32].

### RLRs in MCs

Scarce data indicate that BMMCs and CBMCs express RLRs, and these observations were confirmed upon virus infection. Fukuda et al. [80] observed that resting mouse BMMCs express mRNA for MDA5 and RIG-I, and transcript levels of each receptor increased notably upon vesicular stomatitis virus (VSV) infection. Receptor expression was also established at the protein level by western blotting. Consistent with mRNA results, increased expression of MDA5 and RIG-I protein was noted upon VSV infection. To determine the receptors responsible for the recognition and activation of MCs after VSV infection, BMMCs were treated with siRNA for the particular molecules and examined the antiviral cytokine responses. siRNA inhibition of MDA5 and RIG-I significantly reduced the level of IFN-α, IFN-β, and IL-6 production by MCs in response to VSV. This specific knockdown of each receptor confirmed the contribution of MDA5 and RIG-I in MC anti-VSV defense. Additionally, Sendai virus infection of mature human MCs resulted in a potent up-regulation of RIG-I and MDA-5 mRNA expression that was noticeable after 4 h and was increased further upon 20 h after the onset of infection [81]. Furthermore, stimulation of human MCs by IFN-β strongly up-regulated the expression of MDA-5 and RIG-I. In turn, Brown et al. [82] stated that mRNA RIG-I and MDA5 in human CBMCs was increased following dengue virus and dengue-immune sera treatment.

## Concluding remarks

**Fig. 1:**
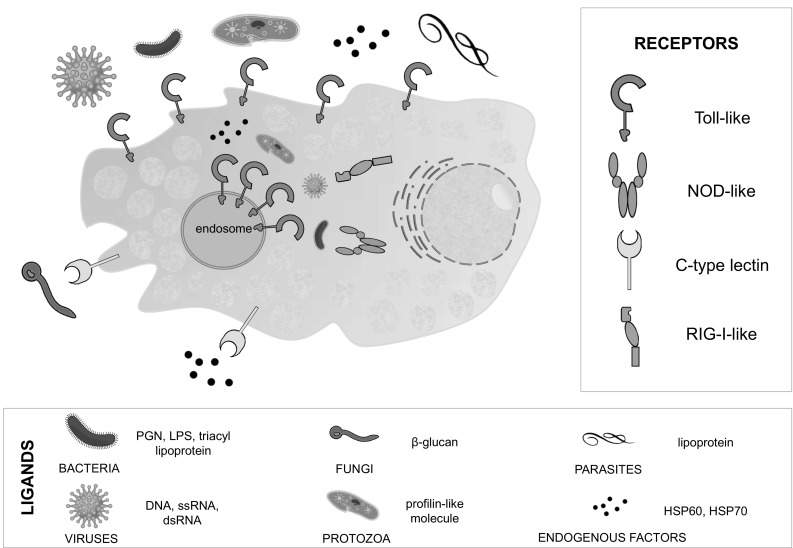
TLR, CLR, NLR and RLR arrangement in MCs: intracellularly in the cytoplasm or in MC membranes, including cell membrane and endosome. Ligand legend contains exemplary ligands derived from a given type of organism/molecule

In summary, hitherto sources assume that MCs, as one of the first line of defense, are adapted to fulfill a wide range of functions in defending against various exogenous intruders or endogenous deleterious factors. Notably, MCs have a broad set of pattern recognition molecules, thus can identify and bind bacterial, viral, fungal, and parasitic PAMPs as well as various endogenous molecules generated in response to infection (Fig. 1). Therefore, MCs may be considered as our internal bodyguards. These abilities allow to conclude that MCs contribute to the control of a wide range of infections and orchestrate mobilization of cells involved in innate and adaptive immune responses.
